# Highlight report: Software for tissue analysis and reconstruction

**DOI:** 10.17179/excli2015-587

**Published:** 2015-09-23

**Authors:** Mohie A. M. Haridy, Yasser S. El-Sayed

**Affiliations:** 1 Department of Pathology and Clinical Pathology, Faculty of Veterinary Medicine, South Valley University, Qena, Egypt; 2 Department of Veterinary Forensic Medicine and Toxicology, Faculty of Veterinary Medicine, Damanhour University, Damanhour, Egypt

## ‬‬‬

Recently, novel image processing and analysis software named TiQuant has been published that allows for reconstruction and quantification of biological tissue from common confocal laser scans (Friebel et al., 2015[5]). Although the software can be applied for analysis of many types of tissue, its reconstruction and analysis methods are specifically tailored to liver tissue. For example, TiQuant permits reconstruction and quantification of the bile canalicular as well as sinusoidal networks of liver lobules. Additionally, a novel 3D surface reconstruction algorithm allows for automated detection, reconstruction and quantification of liver cell boundaries and the three-dimensional shape of liver lobules. TiQuant is able to analyze the volume of hepatocytes, thereby permitting differentiation between mono- and binucleated cells. Moreover, the hepatocyte surface can be analyzed to quantify which fraction of the hepatocyte membrane forms a bile canaliculus or is in contact with a sinusoid. A particular strength of TiQuant is that the position of each cell in relation to larger tissue structures, e.g. central veins or portal veins and bile ducts can be detected, thereby allowing the analysis of individual cell features in relation to lobular position. 

TiQuant opens additional perspectives for spatio-temporal modelling, a discipline of systems biology (Drasdo, 2014[4][3]; Widera, 2014[18]; Ahmed et al., 2014[1]). Understanding the principles and mechanisms that orchestrate the complex coordination of individual cells in an organ requires quantitative 3D and time-resolved analysis of all cells in a tissue (Hammad et al., 2014[10]). 

Previous studies in this field combining image analysis and spatio-temporal modeling have for example identified a previously not recognized order principle by showing that the endothelial cells of the liver sinusoids represent major guide rails for hepatocytes during liver regeneration (Höhme et al., 2007[13]; Hoehme et al., 2010[12]). Moreover, spatio-temporal models have been integrated with metabolic models, and predicted the metabolic performance of healthy and damaged tissues (Schliess et al., 2014[16]). Spatio-temporal models also represent a promising concept to extrapolate between *in vitro* systems and the *in vivo* situation (Godoy et al., 2013[7]; Heise et al., 2012[11]; Mielke et al., 2011[14]; Braeuning et al., 2010[2]; Schug et al., 2013[17]; Ghallab, 2013[6]; Hammad, 2013[8]; Hammad and Ahmed, 2014[9]; Reif et al., 2015[15]). TiQuant will be a valuable tool in this field of research and will facilitate 3D tissue analysis. It is freely available for non-commercial use at http://www.msysbio.com/tiquant. Windows, OSX and Linux are supported.
